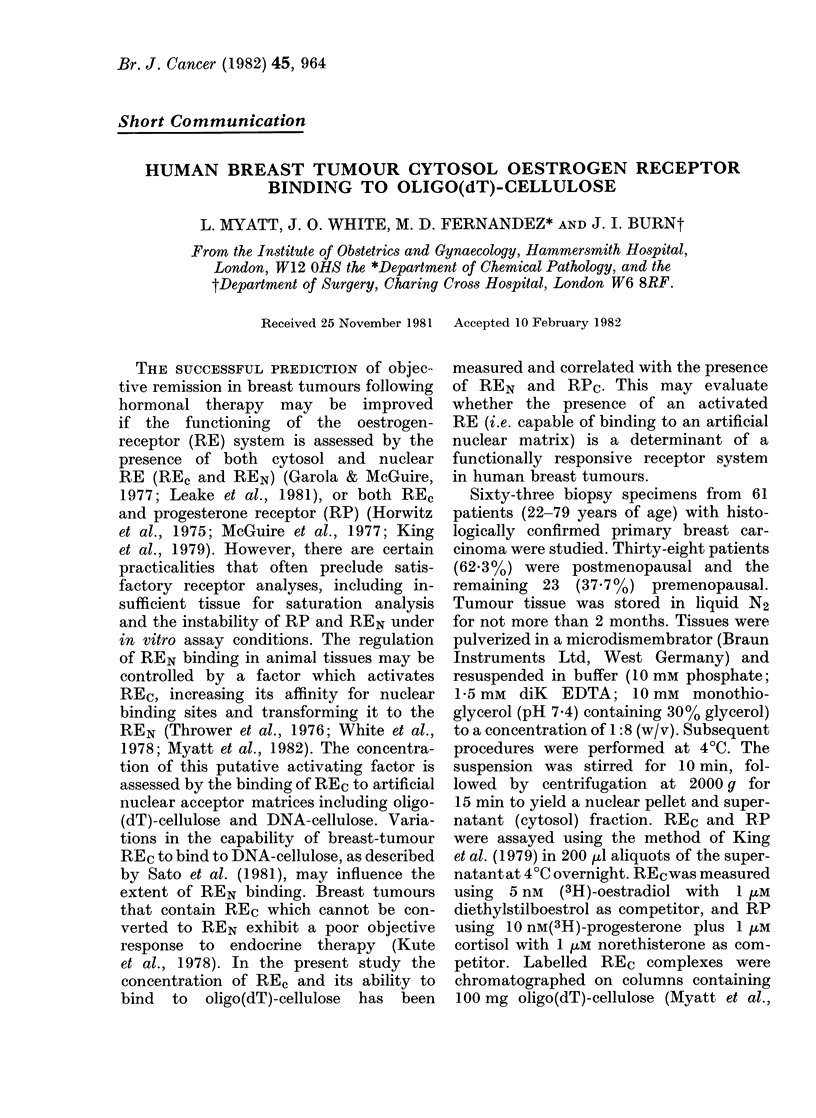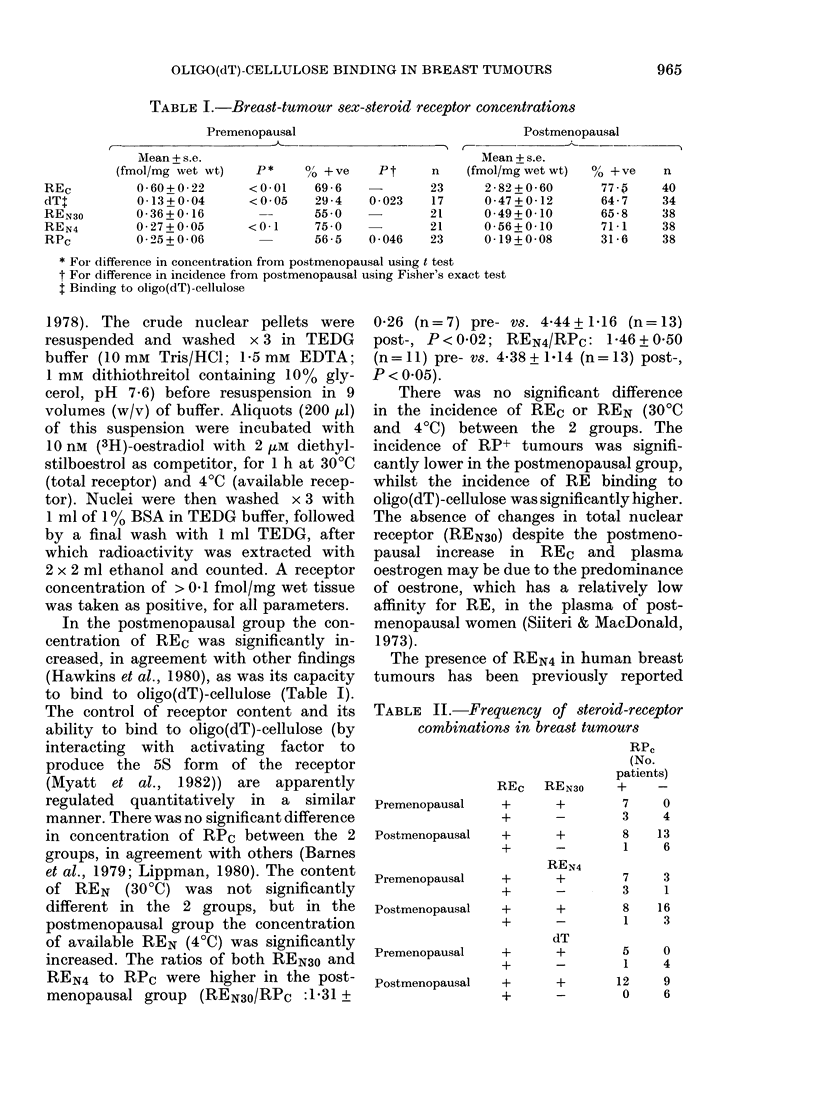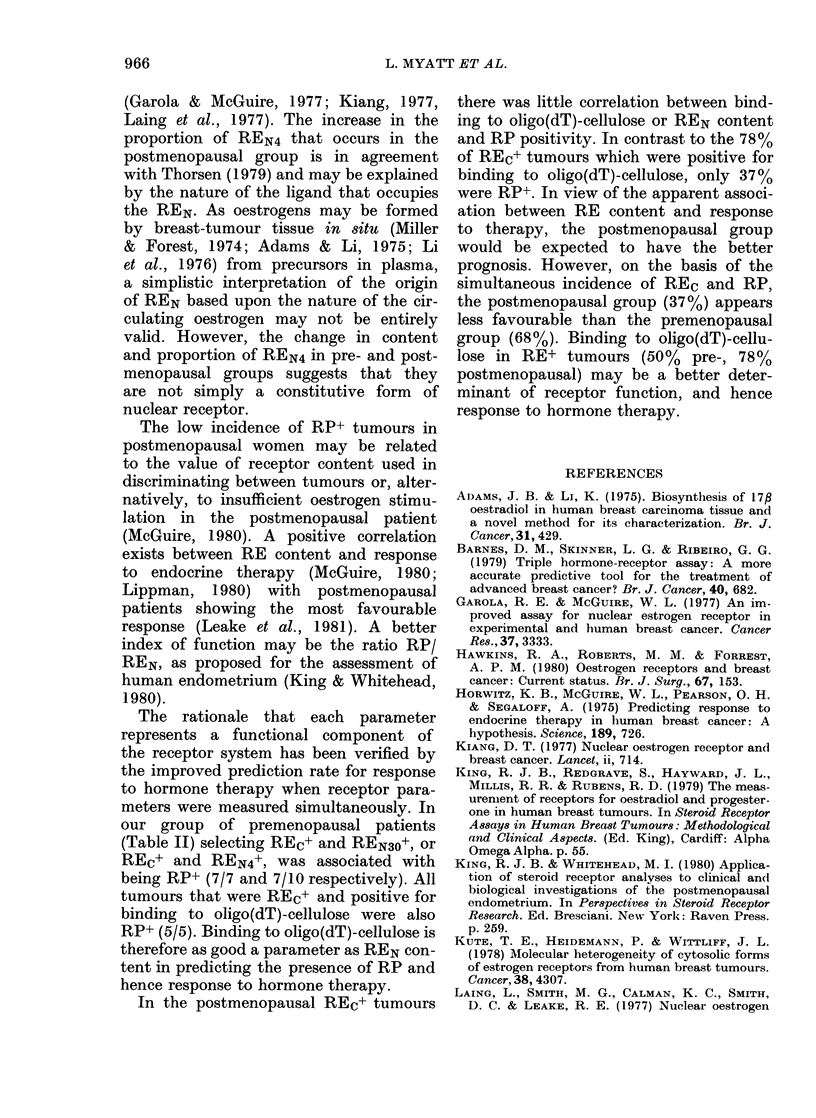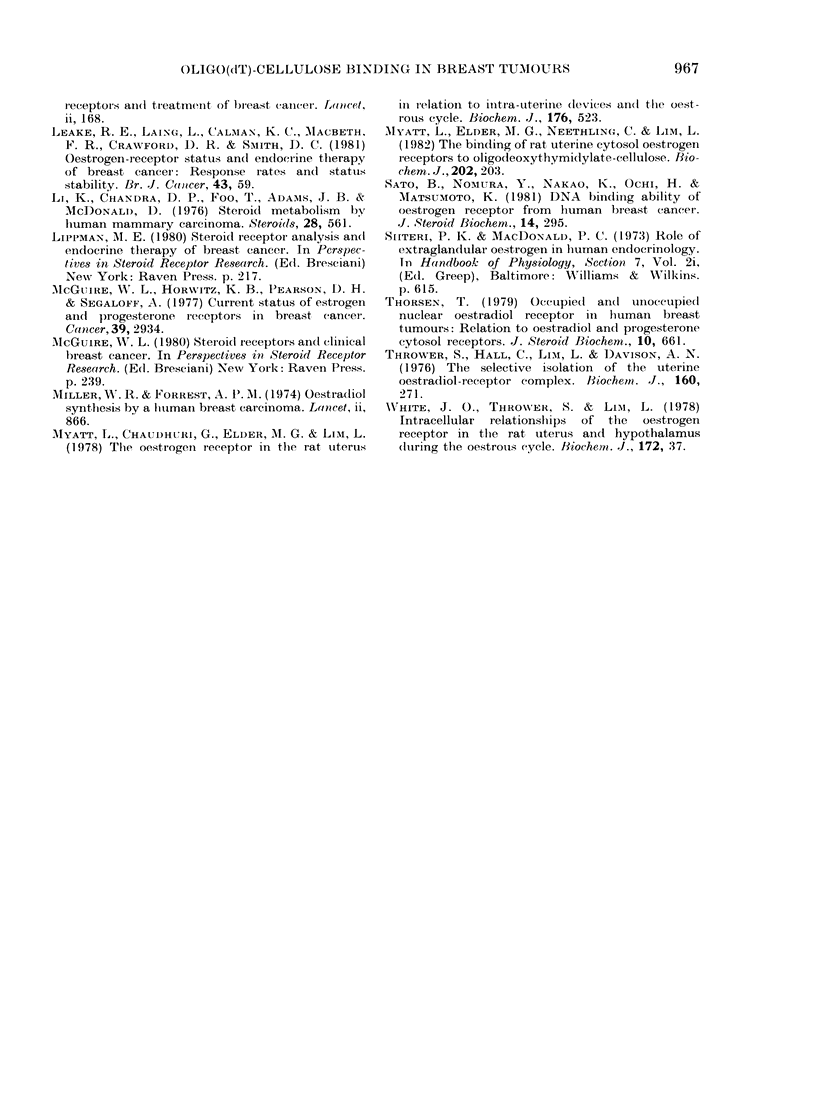# Human breast tumour cytosol oestrogen receptor binding to oligo(dT)-cellulose.

**DOI:** 10.1038/bjc.1982.149

**Published:** 1982-06

**Authors:** L. Myatt, J. O. White, M. D. Fernandez, J. I. Burn


*Br. J. Cancer* (1982) **45**, 964

***Short Communication***

### HUMAN BREAST TUMOUR CYTOSOL OESTROGEN RECEPTOR BINDING TO OLIGO(dT)-CELLULOSE

L. MYATT, J. O. WHITE, M. D. FERNANDEZ* AND J. I. BURN†

*From the Institute of Obstetrics and Gynaecology, Hammersmith Hospital, London, W12 0HS the *Department of Chemical Pathology, and the †Department of Surgery, Charing Cross Hospital, London W6 8RF.*

Received 25 November 1981 Accepted 10 February 1982

THE SUCCESSFUL PREDICTION of objective remission in breast tumours following hormonal therapy may be improved if the functioning of the oestrogen-receptor (RE) system is assessed by the presence of both cytosol and nuclear RE ($RE_c$ and $RE_N$) (Garola & McGuire, 1977; Leake *et al.*, 1981), or both $RE_c$ and progesterone receptor (RP) (Horwitz *et al.*, 1975; McGuire *et al.*, 1977; King *et al.*, 1979). However, there are certain practicalities that often preclude satisfactory receptor analyses, including insufficient tissue for saturation analysis and the instability of RP and $RE_N$ under *in vitro* assay conditions. The regulation of $RE_N$ binding in animal tissues may be controlled by a factor which activates $RE_C$, increasing its affinity for nuclear binding sites and transforming it to the $RE_N$ (Thrower *et al.*, 1976; White *et al.*, 1978; Myatt *et al.*, 1982). The concentration of this putative activating factor is assessed by the binding of $RE_C$ to artificial nuclear acceptor matrices including oligo-(dT)-cellulose and DNA-cellulose. Variations in the capability of breast-tumour $RE_C$ to bind to DNA-cellulose, as described by Sato *et al.* (1981), may influence the extent of $RE_N$ binding. Breast tumours that contain $RE_C$ which cannot be converted to $RE_N$ exhibit a poor objective response to endocrine therapy (Kute *et al.*, 1978). In the present study the concentration of $RE_c$ and its ability to bind to oligo(dT)-cellulose has been measured and correlated with the presence of $RE_N$ and $RP_C$. This may evaluate whether the presence of an activated RE (*i.e.* capable of binding to an artificial nuclear matrix) is a determinant of a functionally responsive receptor system in human breast tumours.

Sixty-three biopsy specimens from 61 patients (22–79 years of age) with histologically confirmed primary breast carcinoma were studied. Thirty-eight patients (62·3%) were postmenopausal and the remaining 23 (37·7%) premenopausal. Tumour tissue was stored in liquid $N_2$ for not more than 2 months. Tissues were pulverized in a microdismembrator (Braun Instruments Ltd, West Germany) and resuspended in buffer (10 mM phosphate; 1·5 mM diK EDTA; 10 mM monothioglycerol (pH 7·4) containing 30% glycerol) to a concentration of 1:8 (w/v). Subsequent procedures were performed at 4°C. The suspension was stirred for 10 min, followed by centrifugation at 2000 *g* for 15 min to yield a nuclear pellet and supernatant (cytosol) fraction. $RE_C$ and RP were assayed using the method of King *et al.* (1979) in 200 μl aliquots of the supernatant at 4°C overnight. $RE_C$ was measured using 5 nM ($^3$H)-oestradiol with 1 μM diethylstilboestrol as competitor, and RP using 10 nM($^3$H)-progesterone plus 1 μM cortisol with 1 μM norethisterone as competitor. Labelled $RE_C$ complexes were chromatographed on columns containing 100 mg oligo(dT)-cellulose (Myatt *et al.*,

TABLE I.—*Breast-tumour sex-steroid receptor concentrations*

| | Premenopausal | | | | | Postmenopausal | | |
|---|---|---|---|---|---|---|---|---|
| | Mean ± s.e. (fmol/mg wet wt) | $P$* | % +ve | $P$† | n | Mean ± s.e. (fmol/mg wet wt) | % +ve | n |
| $RE_C$ | 0·60 ± 0·22 | < 0·01 | 69·6 | — | 23 | 2·82 ± 0·60 | 77·5 | 40 |
| dT‡ | 0·13 ± 0·04 | < 0·05 | 29·4 | 0·023 | 17 | 0·47 ± 0·12 | 64·7 | 34 |
| $RE_{N30}$ | 0·36 ± 0·16 | — | 55·0 | — | 21 | 0·49 ± 0·10 | 65·8 | 38 |
| $RE_{N4}$ | 0·27 ± 0·05 | < 0·1 | 75·0 | — | 21 | 0·56 ± 0·10 | 71·1 | 38 |
| $RP_C$ | 0·25 ± 0·06 | — | 56·5 | 0·046 | 23 | 0·19 ± 0·08 | 31·6 | 38 |

* For difference in concentration from postmenopausal using $t$ test
† For difference in incidence from postmenopausal using Fisher's exact test
‡ Binding to oligo(dT)-cellulose

1978). The crude nuclear pellets were resuspended and washed ×3 in TEDG buffer (10 mM Tris/HCl; 1·5 mM EDTA; 1 mM dithiothreitol containing 10% glycerol, pH 7·6) before resuspension in 9 volumes (w/v) of buffer. Aliquots (200 μl) of this suspension were incubated with 10 nM ($^3$H)-oestradiol with 2 μM diethylstilboestrol as competitor, for 1 h at 30°C (total receptor) and 4°C (available receptor). Nuclei were then washed ×3 with 1 ml of 1% BSA in TEDG buffer, followed by a final wash with 1 ml TEDG, after which radioactivity was extracted with 2×2 ml ethanol and counted. A receptor concentration of >0·1 fmol/mg wet tissue was taken as positive, for all parameters.

In the postmenopausal group the concentration of $RE_C$ was significantly increased, in agreement with other findings (Hawkins *et al.*, 1980), as was its capacity to bind to oligo(dT)-cellulose (Table I). The control of receptor content and its ability to bind to oligo(dT)-cellulose (by interacting with activating factor to produce the 5S form of the receptor (Myatt *et al.*, 1982)) are apparently regulated quantitatively in a similar manner. There was no significant difference in concentration of $RP_C$ between the 2 groups, in agreement with others (Barnes *et al.*, 1979; Lippman, 1980). The content of $RE_N$ (30°C) was not significantly different in the 2 groups, but in the postmenopausal group the concentration of available $RE_N$ (4°C) was significantly increased. The ratios of both $RE_{N30}$ and $RE_{N4}$ to $RP_C$ were higher in the postmenopausal group ($RE_{N30}/RP_C$ : 1·31 ± 0·26 (n = 7) pre- *vs.* 4·44 ± 1·16 (n = 13) post-, $P < 0·02$; $RE_{N4}/RP_C$: 1·46 ± 0·50 (n = 11) pre- *vs.* 4·38 ± 1·14 (n = 13) post-, $P < 0·05$).

There was no significant difference in the incidence of $RE_C$ or $RE_N$ (30°C and 4°C) between the 2 groups. The incidence of $RP^+$ tumours was significantly lower in the postmenopausal group, whilst the incidence of RE binding to oligo(dT)-cellulose was significantly higher. The absence of changes in total nuclear receptor ($RE_{N30}$) despite the postmenopausal increase in $RE_C$ and plasma oestrogen may be due to the predominance of oestrone, which has a relatively low affinity for RE, in the plasma of postmenopausal women (Siiteri & MacDonald, 1973).

The presence of $RE_{N4}$ in human breast tumours has been previously reported

TABLE II.—*Frequency of steroid-receptor combinations in breast tumours*

| | $RE_C$ | $RE_{N30}$ | $RP_c$ (No. patients) + | − |
|---|---|---|---|---|
| Premenopausal | + | + | 7 | 0 |
| | + | − | 3 | 4 |
| Postmenopausal | + | + | 8 | 13 |
| | + | − | 1 | 6 |
| | | $RE_{N4}$ | | |
| Premenopausal | + | + | 7 | 3 |
| | + | − | 3 | 1 |
| Postmenopausal | + | + | 8 | 16 |
| | + | − | 1 | 3 |
| | | dT | | |
| Premenopausal | + | + | 5 | 0 |
| | + | − | 1 | 4 |
| Postmenopausal | + | + | 12 | 9 |
| | + | − | 0 | 6 |

(Garola & McGuire, 1977; Kiang, 1977, Laing *et al.*, 1977). The increase in the proportion of $RE_{N4}$ that occurs in the postmenopausal group is in agreement with Thorsen (1979) and may be explained by the nature of the ligand that occupies the $RE_N$. As oestrogens may be formed by breast-tumour tissue *in situ* (Miller & Forest, 1974; Adams & Li, 1975; Li *et al.*, 1976) from precursors in plasma, a simplistic interpretation of the origin of $RE_N$ based upon the nature of the circulating oestrogen may not be entirely valid. However, the change in content and proportion of $RE_{N4}$ in pre- and postmenopausal groups suggests that they are not simply a constitutive form of nuclear receptor.

The low incidence of $RP^+$ tumours in postmenopausal women may be related to the value of receptor content used in discriminating between tumours or, alternatively, to insufficient oestrogen stimulation in the postmenopausal patient (McGuire, 1980). A positive correlation exists between RE content and response to endocrine therapy (McGuire, 1980; Lippman, 1980) with postmenopausal patients showing the most favourable response (Leake *et al.*, 1981). A better index of function may be the ratio $RP/RE_N$, as proposed for the assessment of human endometrium (King & Whitehead, 1980).

The rationale that each parameter represents a functional component of the receptor system has been verified by the improved prediction rate for response to hormone therapy when receptor parameters were measured simultaneously. In our group of premenopausal patients (Table II) selecting $RE_C^+$ and $RE_{N30}^+$, or $RE_C^+$ and $RE_{N4}^+$, was associated with being $RP^+$ (7/7 and 7/10 respectively). All tumours that were $RE_C^+$ and positive for binding to oligo(dT)-cellulose were also $RP^+$ (5/5). Binding to oligo(dT)-cellulose is therefore as good a parameter as $RE_N$ content in predicting the presence of RP and hence response to hormone therapy.

In the postmenopausal $RE_C^+$ tumours there was little correlation between binding to oligo(dT)-cellulose or $RE_N$ content and RP positivity. In contrast to the 78% of $RE_C^+$ tumours which were positive for binding to oligo(dT)-cellulose, only 37% were $RP^+$. In view of the apparent association between RE content and response to therapy, the postmenopausal group would be expected to have the better prognosis. However, on the basis of the simultaneous incidence of $RE_C$ and RP, the postmenopausal group (37%) appears less favourable than the premenopausal group (68%). Binding to oligo(dT)-cellulose in $RE^+$ tumours (50% pre-, 78% postmenopausal) may be a better determinant of receptor function, and hence response to hormone therapy.

### REFERENCES

Adams, J. B. & Li, K. (1975). Biosynthesis of 17β oestradiol in human breast carcinoma tissue and a novel method for its characterization. *Br. J. Cancer*, **31**, 429.

Barnes, D. M., Skinner, L. G. & Ribeiro, G. G. (1979) Triple hormone-receptor assay: A more accurate predictive tool for the treatment of advanced breast cancer? *Br. J. Cancer*, **40**, 682.

Garola, R. E. & McGuire, W. L. (1977) An improved assay for nuclear estrogen receptor in experimental and human breast cancer. *Cancer Res.*, **37**, 3333.

Hawkins, R. A., Roberts, M. M. & Forrest, A. P. M. (1980) Oestrogen receptors and breast cancer: Current status. *Br. J. Surg.*, **67**, 153.

Horwitz, K. B., McGuire, W. L., Pearson, O. H. & Segaloff, A. (1975) Predicting response to endocrine therapy in human breast cancer: A hypothesis. *Science*, **189**, 726.

Kiang, D. T. (1977) Nuclear oestrogen receptor and breast cancer. *Lancet*, ii, 714.

King, R. J. B., Redgrave, S., Hayward, J. L., Millis, R. R. & Rubens, R. D. (1979) The measurement of receptors for oestradiol and progesterone in human breast tumours. In *Steroid Receptor Assays in Human Breast Tumours: Methodological and Clinical Aspects.* (Ed. King), Cardiff: Alpha Omega Alpha. p. 55.

King, R. J. B. & Whitehead, M. I. (1980) Application of steroid receptor analyses to clinical and biological investigations of the postmenopausal endometrium. In *Perspectives in Steroid Receptor Research.* Ed. Bresciani. New York: Raven Press. p. 259.

Kute, T. E., Heidemann, P. & Wittliff, J. L. (1978) Molecular heterogeneity of cytosolic forms of estrogen receptors from human breast tumours. *Cancer*, **38**, 4307.

Laing, L., Smith, M. G., Calman, K. C., Smith, D. C. & Leake, R. E. (1977) Nuclear oestrogen receptors and treatment of breast cancer. *Lancet*, ii, 168.
LEAKE, R. E., LAING, L., CALMAN, K. C., MACBETH, F. R., CRAWFORD, D. R. & SMITH, D. C. (1981) Oestrogen-receptor status and endocrine therapy of breast cancer: Response rates and status stability. *Br. J. Cancer*, **43,** 59.
LI, K., CHANDRA, D. P., FOO, T., ADAMS, J. B. & McDONALD, D. (1976) Steroid metabolism by human mammary carcinoma. *Steroids*, **28,** 561.
LIPPMAN, M. E. (1980) Steroid receptor analysis and endocrine therapy of breast cancer. In *Perspectives in Steroid Receptor Research.* (Ed. Bresciani) New York: Raven Press. p. 217.
McGUIRE, W. L., HORWITZ, K. B., PEARSON, D. H. & SEGALOFF, A. (1977) Current status of estrogen and progesterone receptors in breast cancer. *Cancer*, **39,** 2934.
McGUIRE, W. L. (1980) Steroid receptors and clinical breast cancer. In *Perspectives in Steroid Receptor Research.* (Ed. Bresciani) New York: Raven Press. p. 239.
MILLER, W. R. & FORREST, A. P. M. (1974) Oestradiol synthesis by a human breast carcinoma. *Lancet*, ii, 866.
MYATT, L., CHAUDHURI, G., ELDER, M. G. & LIM, L. (1978) The oestrogen receptor in the rat uterus in relation to intra-uterine devices and the oestrous cycle. *Biochem. J.*, **176,** 523.
MYATT, L., ELDER, M. G., NEETHLING, C. & LIM, L. (1982) The binding of rat uterine cytosol oestrogen receptors to oligodeoxythymidylate-cellulose. *Biochem. J.*, **202,** 203.
SATO, B., NOMURA, Y., NAKAO, K., OCHI, H. & MATSUMOTO, K. (1981) DNA binding ability of oestrogen receptor from human breast cancer. *J. Steroid Biochem.*, **14,** 295.
SIITERI, P. K. & MACDONALD, P. C. (1973) Role of extraglandular oestrogen in human endocrinology. In *Handbook of Physiology, Section* 7, Vol. 2i, (Ed. Greep), Baltimore: Williams & Wilkins. p. 615.
THORSEN, T. (1979) Occupied and unoccupied nuclear oestradiol receptor in human breast tumours: Relation to oestradiol and progesterone cytosol receptors. *J. Steroid Biochem.*, **10,** 661.
THROWER, S., HALL, C., LIM, L. & DAVISON, A. N. (1976) The selective isolation of the uterine oestradiol-receptor complex. *Biochem. J.*, **160,** 271.
WHITE, J. O., THROWER, S. & LIM, L. (1978) Intracellular relationships of the oestrogen receptor in the rat uterus and hypothalamus during the oestrous cycle. *Biochem. J.*, **172,** 37.